# Merkel cell polyomavirus and cutaneous Merkel cell carcinoma

**DOI:** 10.4155/fsoa-2016-0064

**Published:** 2016-11-16

**Authors:** Ettore Minutilli, Antonino Mulè

**Affiliations:** 1 Catholic University of the Sacred Heart, Milan, Italy; 2 Fondazione Policlinico ‘Agostino Gemelli’ – Largo A. Gemelli, 8 – 00168 – Roma, Italia

**Keywords:** circulating biomarkers, immunotherapy, Merkel cell carcinoma, Merkel cell polyomavirus, sentinel node biopsy

Merkel cell carcinoma (MCC) is a rare aggressive neuroendocrine skin cancer of elderly patients. Generally, it originates from Merkel cells of the basal layer of the epidermis characterized by typical neuroendocrine granules (neuron-specific enolase; NSE+) and keratin filament (cytokeratin-20; CK20^+^). Its incidence has been increasing in the last two decades, likely because of improved ability to diagnose it. However, its mortality is still too high owing to systemic metastases (20–30% of MCC) despite the early detection of locally advanced disease through sentinel node (SN) biopsy and standard therapies [1].

Common risk factors for developing MCC include advanced age and immunocompromised state (secondary neoplasm, organ transplantation, HIV infection, drug-induced state and so on) and chronic UV exposure.

Histopathologically, the most important prognostic indicators are represented by size, thickness, depth, mitotic rate, lymphovascular invasion and tumoral infiltrating CD8^+^ T cells (tumoral infiltration of lymphocytes [TIL]) of the primary MCC as well as SN status.

More recently, a human polyomavirus (MCPyV) has been discovered as an oncogenic agent of MCC [2]. It is a small, circular double-stranded DNA virus of the family Polyomaviridae. MCPyV sequences have revealed a very low genetic variability with the presence of five major variants corresponding to the different continents [3]. However, MCC is frequently reported in patients of Caucasian origin, mainly North America and Europe including Italy, while rarely in Asia and exceptionally in Africa.

To date, there is a robust collection of scientific evidence supporting its classification as a causative agent of MCC according to the WHO’s International Agency for Research on Cancer; however, a combination of this potential oncogenic pathway with other clinical factors and particularly immunosuppression seems to be mandatory for the pathogenesis of this rare skin cancer. In fact, MCPyV infection is common in the human population and skin is the most frequent asymptomatic location. Moreover, up to 80% of the adult population contains serum antibodies to the major capsid protein, VP1. However, the clonal integration of its viral genome into Merkel cells, and most frequently on chromosome 5, can induce mutations of the early region that result in truncation of the large T antigen (LTAg) inactivating pRB tumor suppressor function, while the small T antigen (STAg) coding sequence remains generally intact but promotes translation, instead. These MCC tumor-specific mutations abrogate viral DNA replication capacity and subsequently cell death, but preserve its oncogenic function with induction of uncontrolled cellular proliferation [4].

MCPyV LTAg and DNA have been detected by immunohistochemistry and PCR, respectively, on formalin-fixed paraffin-embedded tissue samples in 80–100% of MCC; on the contrary, there are conflicting results on the rates of MCPyV in tumoral (non-MCC) and normal skin or other cancers [5–8].

Recent studies have supported the model that MCPyV contributes to the pathogenesis of most MCC; however, in reality, different oncologic pathways may be responsible for the development of MCPyV-negative MCC. In fact, while MCC incidence is very low, seroprevalence for the virus is high, which would suggest that infection by the virus is very common. Thus, the virus might be considered restrained whereas, as a matter of fact, it persists in an asymptomatic state that can only occasionally be disrupted to lead to neoplastic progression towards MCC. In addition, there are other factors that can induce genetic mutations such as TP53, PIK3CA and so on in MCPyV-negative MCC [9,10].

Moreover, MCPyV-positive MCCs are generally characterized by standard differentiation (CK20^+^) and high immunological response (TIL and peripheral blood specific CD8^+^ lymphocytes as well as serum anti-MCPyV antibodies titer) with favorable prognosis. In fact, high TIL and particularly CD8^+^ seems to greatly influence the population of MCC cells killing tumoral cells through cytotoxic mechanisms with better survival [11]. Besides, high titers of MCPyV antibodies and particularly anti-LTAg, but above all circulating specific CD8^+^ cells, seem to be associated with better survival. On the contrary, different oncologic pathways seem to be responsible for the development of uncommon MCPyV-negative MCC [12,13], which are frequently characterized by divergent differentiation and low immunological response with poor prognosis [14].

The management of MCC remains challenging following this new knowledge into its molecular biology [15]; this is somewhat analogous to oropharyngeal cancer following the identification of human papillomavirus as a causative agent. Further studies are required to fully resolve the prognostic significance of MCPyV [16] in future MCC staging systems as well as its clinical and therapeutic implications. In fact, the correlation of MCPyV-positive/negative MCC with other common prognostic factors and particularly SN status might eventually clarify the independent prognostic value of MCPyV [6,17], confirming the different pathogenetic pathways of the two variants [9,18].

According to the EORTC-SPECTA and US-based NCI-MATCH programmes, clinical trials using novel targeted and immunotherapies are currently under investigation; circulating tumor cells might serve as biomarkers for disease surveillance in agreement with antitumoral activity (anti-MCPyV CD8^+^ T cells rather than antibodies) [19,20]. MCPyV-positive tumors as well as these infiltrating and circulating specific T cells seem to express respectively higher levels of PD-L1 and PD-1 [21,22] in comparison with MCPyV-negative ones. Therefore, anti-PD-L1 or anti-PD-1 immunotherapies, preferably in combination with molecular targeted treatment, might become the gold standard for metastatic disease, independent of the presence of the virus [23]. In fact, both radiation and chemotherapies have demonstrated poor results in the adjuvant setting in terms of regional control and survival benefit, respectively, with high toxicity in elderly patients, while adjuvant immunotherapy (anti-PD-1 or anti-PD-L1 rather than anti-CTLA-4) might be confirmed more efficacious with acceptable toxicity [23,24]. Alternative immunotherapy-based treatments (vaccines, interferon, interleukins and so on) are also being tested [25–27].
